# How the Processing Mode Influences Azure Kinect Body Tracking Results

**DOI:** 10.3390/s23020878

**Published:** 2023-01-12

**Authors:** Linda Büker, Vincent Quinten, Michel Hackbarth, Sandra Hellmers, Rebecca Diekmann, Andreas Hein

**Affiliations:** 1 Assistance Systems and Medical Device Technology, Department for Health Services Research, School of Medicine and Health Sciences, Carl von Ossietzky University, Ammerländer Heerstraße 114-118, 26129 Oldenburg, Germany; 2 Geriatric Medicine, Department for Health Services Research, School of Medicine and Health Sciences, Carl von Ossietzky University, Ammerländer Heerstraße 114-118, 26129 Oldenburg, Germany

**Keywords:** Azure Kinect, body tracking, skeleton tracking, Azure Kinect Body Tracking SDK, reproducibility, quality assurance

## Abstract

The Azure Kinect DK is an RGB-D-camera popular in research and studies with humans. For good scientific practice, it is relevant that Azure Kinect yields consistent and reproducible results. We noticed the yielded results were inconsistent. Therefore, we examined 100 body tracking runs per processing mode provided by the Azure Kinect Body Tracking SDK on two different computers using a prerecorded video. We compared those runs with respect to spatiotemporal progression (spatial distribution of joint positions per processing mode and run), derived parameters (bone length), and differences between the computers. We found a previously undocumented converging behavior of joint positions at the start of the body tracking. Euclidean distances of joint positions varied clinically relevantly with up to 87 mm between runs for CUDA and TensorRT; CPU and DirectML had no differences on the same computer. Additionally, we found noticeable differences between two computers. Therefore, we recommend choosing the processing mode carefully, reporting the processing mode, and performing all analyses on the same computer to ensure reproducible results when using Azure Kinect and its body tracking in research. Consequently, results from previous studies with Azure Kinect should be reevaluated, and until then, their findings should be interpreted with caution.

## 1. Introduction

Reproducibility is one of the main quality criteria in research. Measurement errors of instruments should be small and stable to ensure reproducible and consistent results. Like any other measurement instrument, popular depth cameras do not provide error-free measurements. A frequently used depth camera in research is the Microsoft Azure Kinect DK RGB-D-camera with its Azure Kinect Body Tracking SDK. Among other things, it is used for movement analysis [1,2] as well as posture analysis [3,4]. The Azure Kinect has a 1-megapixel time-of-flight (ToF) camera installed for depth measurement, which has a typical systematic error of <11 mm + 0.1% of the distance to the object and a random error with a standard deviation of ≤17 mm according to its manufacturer [5]. These numbers were confirmed in a study by Kurillo et al. [6]. The systematic error describes the difference between the measured depth (time average over several frames) and the correct depth. Multi-path interference, i.e., the condition when a sensor pixel integrates light reflected from multiple objects, is not considered here. The random error, on the other hand, is the standard deviation of the depth over time [7]. One source of depth noise is introduced by the internal temperature of the depth sensor. Tölgyessy et al. found that the camera needed to be warmed up for about 50–60 min before a stable depth output was acquired [8].

Not only does the sensory system itself introduces measurement error, all elements in the entire processing chain, such as the body tracking using Microsoft’s Azure Kinect Body Tracking SDK [9], introduce additional measurement errors. Measurement errors propagate and might be amplified further up the chain. Albert et al. found a mean Euclidean distance between 10 mm and 60 mm, depending on the tracked joint, in a comparison between the optical marker-based Vicon system (Vicon Motion Systems, Oxford, UK) as the gold standard and the Azure Kinect [10]. Ma et al. found a Root Mean Square Error (RMSE) of the joint angles in the lower extremities between 7.2° and 32.3° compared to the Vicon system [11].

According to Tölgyessy et al., the depth mode of the camera (wide field of view (WFOV) or narrow field of view (NFOV)), as well as the distance of the person to the camera, also have a large influence on the accuracy of the body tracking [12]. They showed a strong increase in the standard deviation by more than 2.2 m from the camera in WFOV and more than 3.7 m in NFOV.

Romeo et al. have shown that ambient light conditions also influence the accuracy of body tracking. They found an up to 2.4 times higher mean distance error when the subject was illuminated with 1750 lux compared to 10 lux [13]. However, both light conditions are not realistic for a study with human subjects. Furthermore, we question whether the halogen light source used by Romeo et al. provided a considerable source of infrared light, which might have influenced the results. Our original goal was to investigate the influences of ambient (natural and artificial) light on body tracking accuracy.

During the initial data analysis of our data collected under different light conditions, we found considerable differences in the body tracking results when running the Azure Kinect Body Tracking SDK using the processing mode CUDA (short for Compute Unified Device Architecture, developed by NVIDIA) repeatedly on the same video. These differences in body tracking mean that results cannot be reproduced, and therefore, quality assurance and good scientific practice are not assured. At first sight, the differences were not explainable by the known sources of measurement error from previous studies described above. These studies focused mainly on the validity of the Kinect’s measurements and its body tracking. In contrast, the differences we observed are signs of a repeatability instead of solely a validity problem. As a consequence, the inconsistent results of repeated body tracking runs may have influenced the validity analyses in previous studies. Additionally, the dimension of the measurement error introduced by repeated body tracking runs might be clinically relevant, although unknown, since we did not find any literature describing differences between multiple runs of the body tracking. Therefore, we decided to perform additional experiments to further investigate the error introduced by the body tracking processing mode before investigating the influence of ambient light. These additional experiments and their results are the focus of this paper. The aim of this paper is to analyze and quantify the effects of the chosen processing mode on the results when using the Azure Kinect DK in combination with repeated body tracking runs using the Azure Kinect Body Tracking SDK.

The structure of this paper is as follows: Section 2 describes the experimental setup as well as the used hardware and software. Section 3.1 presents the methods and results of changes over time. Section 3.2 deals with the methods and results of the comparison of processing modes. Section 3.3 handles the comparison of different computers, followed by the discussion and conclusion in Section 4 and Section 5, respectively.

## 2. Materials

In this paper, we aim to quantify the different results caused by the body tracking’s processing mode. For all analyses, the experimental setup described in Section 2.1 was used together with the hardware and software described in Section 2.2.

### 2.1. Experimental Setup

To analyze the effects of running body tracking multiple times, we created an experimental setup designed to minimize the effects of other sources of noise that might influence the results. Therefore, we recorded a single 30-s video that served as input for all our experiments. The video was recorded in a windowless room without reflective surfaces.

**Figure 1:**
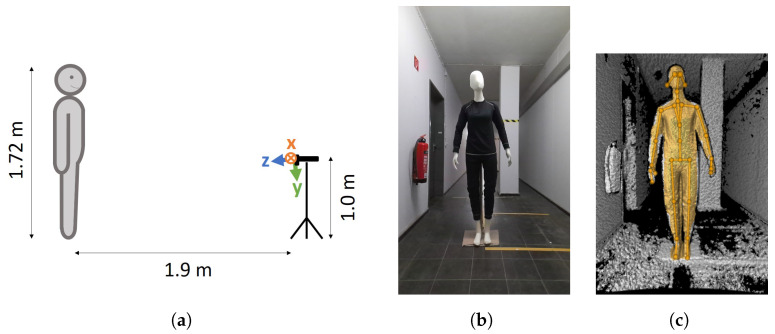
Overview of the experimental setup. (**a**) Schematic setup. In addition, the coordinate system of the depth camera is shown, which is tilted downwards by 6° with respect to the camera’s case. (**b**) Mannequin from the camera’s point of view in a windowless dark room (picture taken with the lights turned on). (**c**) Point cloud of the mannequin with overlaid body tracking. Screenshot of *k4abt_simple_3d_viewer* from the Azure Kinect Body Tracking SDK.

To exclude the influences of external light sources, which might have an effect on the body tracking, as described by Romeo et al. [13], we turned off all lights in the room during the recording. To exclude the effects of movement on the results, we used a mannequin of 1.72 m in height instead of a human (Figure 1). The mannequin was stationary, i.e., did not move during the experiments, and was positioned in a neutral pose such that all joints were within the camera’s field of view without (self-)occlusions (Figure 1b). The mannequin was placed frontally to the camera in a distance of 1.9 m. This distance represents the middle of the optimal range (0.5–3.86 m) for the NFOV of the camera [6,8]. According to the literature, a lateral view of the person is useful when either only the side of the body facing the camera is to be analyzed [14] or the side of the body facing away from the camera occludes itself [15]. However, since there is no occlusion in this study and both sides of the body are to be analyzed equally, the camera was placed frontally to the mannequin. The camera was placed on a tripod in front of the mannequin at approximately 1 m height to ensure a centered position of the mannequin in the depth camera’s field of view. In addition, the camera case was aligned horizontally using a spirit level on top of the camera. A schematic overview of the setup is shown in Figure 1a.

In accordance with the results of Tölgyessy et al. [8], the camera was warmed up for an hour before recording. To check whether body tracking was able to estimate the skeleton of the mannequin, we used the *k4abt_simple_3d_viewer* from the Azure Kinect Body Tracking SDK. As shown in Figure 1c, the mannequin’s skeleton was recognized by the body tracking.

### 2.2. Hardware and Software

The 30-s video was recorded with the Microsoft Azure Kinect DK, using the Azure Kinect Recorder from the Azure Kinect SDK version 1.4.1 and the following parameters:

- Frame rate: 30 frames per second (FPS)
- Depth mode: narrow field of view, unbinned
- Color format: MJPG
- Color resolution: 2048 × 1536 pixels
- RGB camera firmware version: 1.6.110
- Depth camera firmware version: 1.6.80

The Azure Kinect DK depth camera’s coordinate system *x*-axis points to the right, the *y*-axis to the bottom, and the *z*-axis to the front (seen from the camera’s perspective and visualized in Figure 1a). The depth camera is tilted downward by 6° relative to the camera’s RGB-lens and the camera’s case [16].

For the body tracking, we used the recorded video as input for all analyses utilizing the Microsoft Azure Kinect Body Tracking SDK version 1.1.2. The video was processed using the *offline_processor* from the Azure Kinect Samples on GitHub downloaded on 30 June 2022 [17]. The results from the *offline_processor* were stored in a JSON file.

The *offline_processor* was executed 100 times for each of the four processing modes (Central Processing Unit (CPU), Compute Unified Device Architecture (CUDA), Direct Machine Learning (DirectML), and TensorRT) provided by the Azure Kinect Body Tracking SDK. This process was performed on two desktop computers: computer A, with an Intel Core i9-10980XE 18-Core Processor running at 4.80 GHz and NVIDIA GeForce RTX 3080 graphic card; and computer B, with an AMD Ryzen 7 5800X 8-Core Processor running at 3.80 GHz and a NVIDIA GeForce RTX 3070Ti graphic card. Both computers were running Windows 10. In the remainder, we present the results from computer A for all analyses unless stated otherwise. In Section 3.3, we present and compare the results from both computers.

Of the 32 calculated joints provided by the Azure Kinect Body Tracking SDK, we limited our analyses to the set of main joints listed in Table 1. This was performed to provide a better overview of the relevant joints for posture analysis and because the excluded joints are difficult for the camera to recognize accurately. The results of the body tracking were analyzed using Python 3.8.10, except for the calculations of the ellipsoids for which we used MATLAB (version 2022a, The MathWorks Inc., Natick, MA, USA).

## 3. Methods and Results

**Figure 2:**
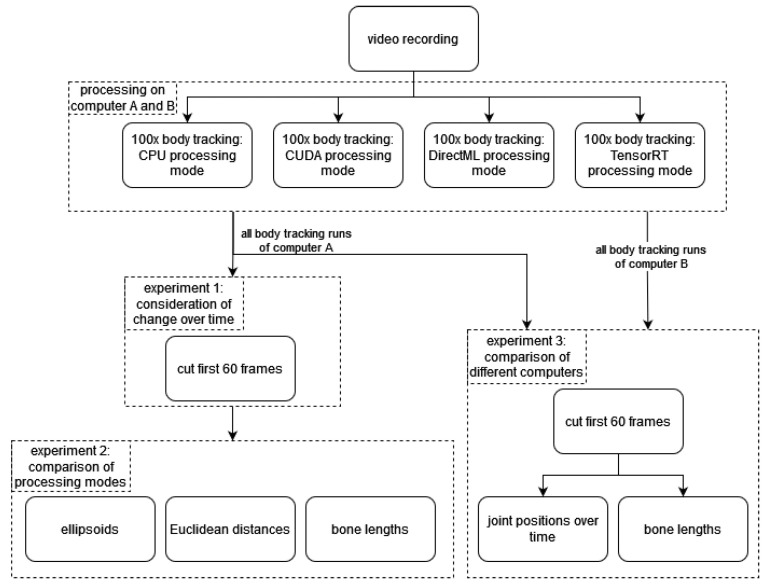
Schematic overview of the data processing and the three experiments.

To analyze the body tracking and their different results by processing mode, three main experiments were conducted. In the first experiment (Section 3.1), we processed the video of the static mannequin multiple times in each processing mode to analyze possible changes in joint positions over time caused by the body tracking. The second experiment (Section 3.2) deals with the effects of the individual processing modes on the joint positions, analyzing both the spatiotemporal distribution and the effect on derived parameters (e.g., bone length). In the last experiment (Section 3.3), these analyses were performed on two computers, and their results were compared. Figure 2 shows a schematic overview of the data processing and the experiments.

### 3.1. Consideration of Change over Time

#### 3.1.1. Methods

To visualize the changes in the body tracking results over time, the included joint positions from Table 1 for the setup described in Section 2 were plotted in relation to the depth sensor’s *x*-, *y*-, and *z*-axes for all body tracking runs and each processing mode.

In the resulting visualization, we noticed a converging behavior of the joint positions in the first few seconds from the start of the body tracking (described in detail below in Section 3.1.2). To model and quantify this converging behavior, we fitted an exponential curve for each axis, processing mode, and body tracking run using Python’s *scipy.optimize.curve_fit* function and the following curve fitting formula:

(1) $$f\left(x\right)=a\cdot e^{-b\cdot x}+c$$

where a,b,c∈R; constant coefficients calculated by Python’s *curve_fit* function, x∈R.

For each of the fitted curves, we calculated the half-life time T12 using the formula:

(2) T12=ln(2)b

with *b* from Equation (1). We considered four times T12 (93.75%) as the cut-off point between the convergence and the random noise in the signal, i.e., considered the end of the convergence at x=4·T12. For each axis, joint, and processing mode, the mean was calculated. The overall cut-off point was calculated using the 85%-quantile over these means.

#### 3.1.2. Results

**Figure 3:**
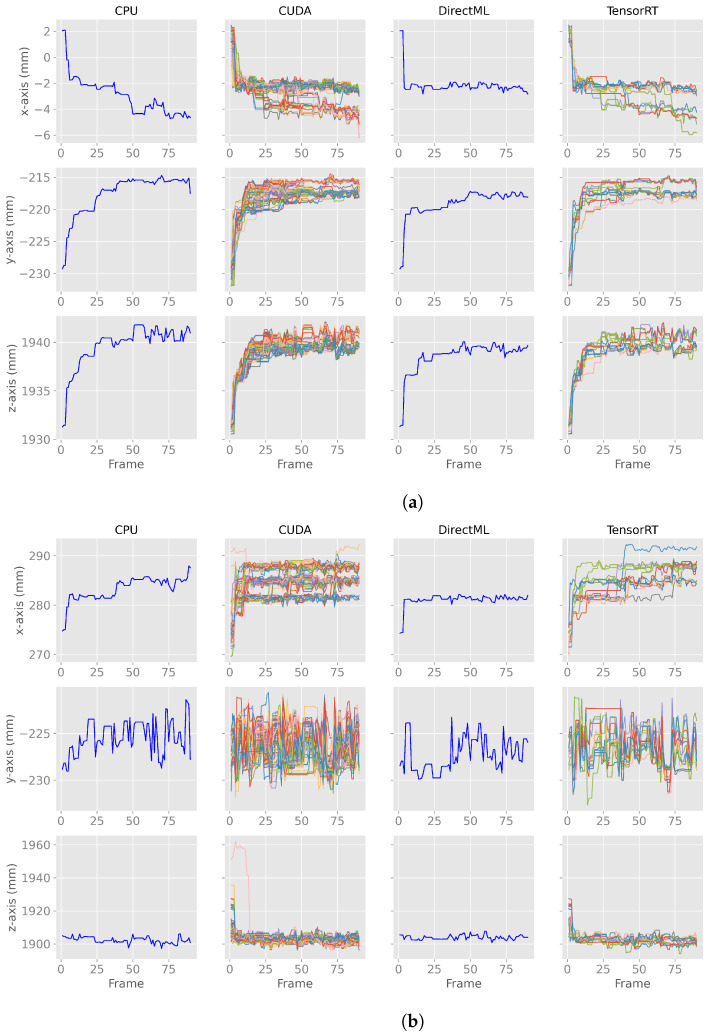
*X*-, *y*-, *z*-axes of the first 90 body tracking frames using 100 body tracking runs for all four processing modes for joint positions of PELVIS and WRIST_LEFT. Note: the runs for CPU and DirectML yielded the same results for each run and, therefore, appear as a single line. (**a**) *X*-, *y*-, *z*-axes of the first 90 body tracking frames for the joint position of PELVIS. (**b**) *X*-, *y*-, *z*-axes of the first 90 body tracking frames for the joint position of WRIST_LEFT.

We observed an undocumented converging initialization behavior of joint positions estimated by the Azure Kinect Body Tracking SDK in the first seconds. Figure 3 shows this behavior for two exemplary joints in the first 90 frames: (a) the PELVIS and (b) the WRIST_LEFT. The PELVIS (Figure 3a) showed similar converging behavior to a steady state for all processing modes and all axes. For other joints, such as the WRIST_LEFT (Figure 3b), this converging behavior took only a few frames (e.g., CPU/DirectML *x*-axis in Figure 3b) or was not that distinctive (e.g., CUDA/TensorRT *y*-axis in Figure 3b). The duration of the converging behavior varied by joint and processing mode and ranged from 2 frames (SPINE_CHEST; DirectML) to 360 frames (FOOT_RIGHT; TensorRT). It is also noticeable that while most of the joints stabilized from left to right (*x*-axis), top to bottom (*y*-axis), and front to back (*z*-axis), some stabilized the other way around, e.g., the *x*-axis of the PELVIS stabilized from right to left. The range of stabilization differed between the various joints, axes, and processing modes. The range of stabilization for the *y*-axis of the WRIST_LEFT for processing mode CPU, for example, was less than 5 mm, while the *z*-axis of the same joint using CUDA showed a converging range of about 60 mm.

**Figure 4:**
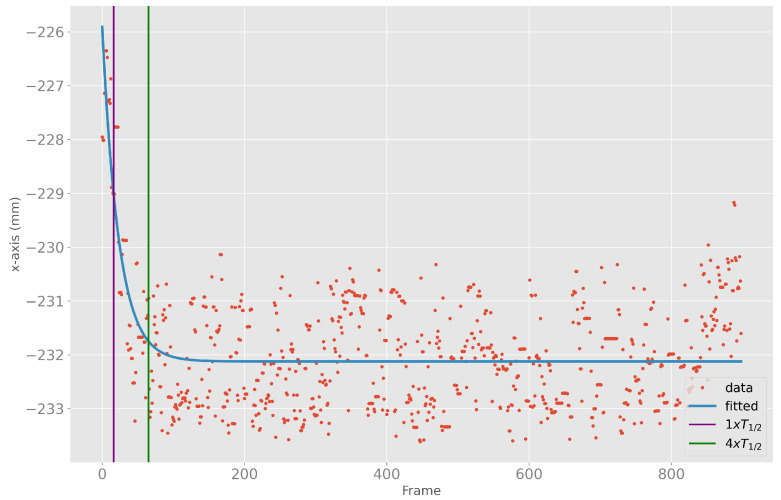
*X*-position data (red) of ELBOW_RIGHT, fitted exponential curve (blue), as well as one (purple) and four (green) times the half-life time of the fitted exponential curve.

The converging initialization behavior at the start of the body tracking can have a large influence on the accuracy of the results. The overall 85%-quantile of four times the half-life time was frame 55. For ease of understanding, Figure 4 shows an example of the *x*-positions of the ELBOW_RIGHT, the fitted exponential curve, the half-life time, and the fourfold half-life time.

### 3.2. Comparison of Processing Modes

#### 3.2.1. Methods

In Section 3.1.2, we observed a previously undocumented converging initialization behavior in the first seconds of the body tracking. Since these partially large variations can have a substantial influence on the quantification of the differences between the processing modes, we decided to discard the data in which the converging behavior was observed. Since the duration of the converging behavior highly varied by joint, axis, and processing mode, we decided to take the 85%-quantile (55 frames; Section 3.1.2) and round it up to the next full second of the video (frame 60, i.e., 2 s @30 FPS). Therefore, the first 60 frames were discarded in all analyses from this point on, i.e., only frames 61 until 900 were included in the analyses.

Differences in body tracking caused by the processing mode were quantified using three different metrics:

(1) Volume of ellipsoids containing positions of one joint each: As the joint positions scatter in all three dimensions, they form a volume. The first observations showed that the scattering was not equally distributed over all axes. Since there was often a dominant direction, an ellipsoid was chosen over a sphere. These ellipsoids were calculated per processing mode and joint (Table 1) using MATLAB and the data of each of the 100 body tracking runs per processing mode. The function *PLOT_GAUSSIAN_ELLIPSOID* written by Gautam Vallabha from the MATLAB central file exchange [19], was used for these calculations. As parameters for this function, we used SD = 2 and NPTS = 20 to create an ellipsoid with 20 faces that encapsulates approximately 86.5% of all data points [20]. The volume of the resulting ellipsoid was then calculated using MATLAB’s *convhull* function by feeding it the data points of the ellipsoid.

(2) Euclidean distance between positions of a joint between the 100 runs: The Euclidean distances $d_{t}\left(p,q\right)$ were calculated using the following formula:

(3) $$d_{t,joint}\left(p_{t,i},q_{t,j}\right)=\sqrt{{\left(p_{t,x_{i}}-q_{t,x_{j}}\right)}^{2}+{\left(p_{t,y_{i}}-q_{t,y_{j}}\right)}^{2}+{\left(p_{t,z_{i}}-q_{t,z_{j}}\right)}^{2}},$$

where *t* = time in frame number 61…900, *joint* ∈ included joints (Table 1), $p_{{\left\{x/y/z\right\}}_{i}}$ = joint position for *joint in i* for *x*/*y*/*z* axes, respectively, $q_{{\left\{x/y/z\right\}}_{j}}$ = joint position for *joint* in *j* for *x*/*y*/*z* axes, respectively, *i* = body tracking run 1…100, *j* = body tracking run 1…100.

The Euclidean distances were calculated for each frame and joint position from one body tracking run to all other runs within the same processing mode, i.e., when *p* was the position of the PELVIS in frame 61 for processing mode CPU in run 1, *q* was the position of the PELVIS in frame 61 for processing mode CPU in run 2 until 100.

(3) Bone length of the extremities: The bone lengths of the extremities were calculated with *s* as the start joint and *e* as the end joint from Table A1 using the following formula:

(4) $$b_{t,bone}\left(s_{t},e_{t}\right)=\sqrt{{\left(s_{t,x}-e_{t,x}\right)}^{2}+{\left(s_{t,y}-e_{t,y}\right)}^{2}+{\left(s_{t,z}-e_{t,z}\right)}^{2}},$$

where *t* = time in frame number 61…900, *bone* ∈ bones (Table A1). $s_{\left\{x/y/z\right\}}$ = position of the start joint (Table A1) for bone for *x*/*y*/*z* axes, respectively, $e_{\left\{x/y/z\right\}}$ = position of the end joint (Table A1) for bone for *x*/*y*/*z* axes, respectively.

The bone lengths were calculated for each frame and processing mode within the same body tracking run.

We calculated and reported the minimum, maximum, mean, median, and standard deviation for each metric, processing mode, and joint or bone length, respectively.

#### 3.2.2. Results

**Figure 5:**
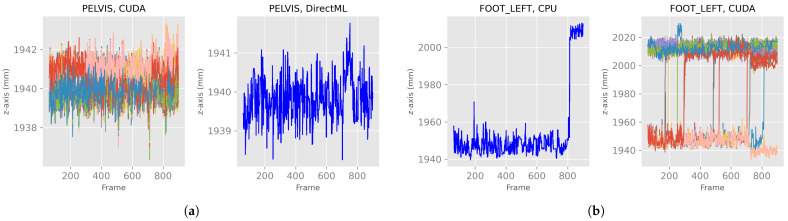
*X*-, *y*-, and *z*-axes of the joint positions of PELVIS and FOOT_LEFT for all four processing modes—extract of relevant plots (Figures for all processing modes and axis are shown in Figure A1). (**a**) Examples of a stable, steady value range. (**b**) Examples of a switch between two steady value ranges.

After discarding the first 60 frames of the body tracking results, the *x*-, *y*-, and *z*-axes of the positions for all four processing modes looked like the exemplary plots for PELVIS and FOOT_LEFT in Figure 5. Note that the converging stabilization phase disappeared. The positions were stable in a distinct value range, i.e., steady state, for example, the *z*-axis of PELVIS for CUDA or DirectML in Figure 5a, or they switched between two (or more) steady value ranges (e.g., *z*-axis of FOOT_LEFT for CPU or CUDA in Figure 5b). Furthermore, it can be seen that while DirectML and CPU showed the same progression of position in the 100 runs, represented by the single line, CUDA and TensorRT showed differences in position during the 100 runs.

##### Distribution of Joint Positions over Time across the Three Axes

**Figure 6:**
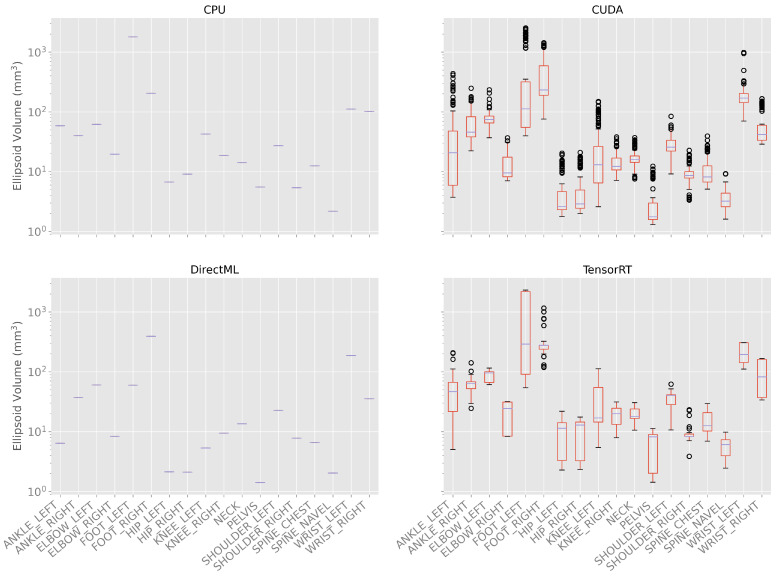
Box plots of the ellipsoid volumes for all processing modes for the 100 body tracking runs. As the standard deviation for processing modes CPU and DirectML is zero, only the mean is shown for these modes. Note: the *y*-axis has a logarithmic scale.

The ellipsoids and their volumes visualize the distribution of the joint positions over time. The volumes of the ellipsoids are shown as box plots in Figure 6 and in Table A2 in the appendix.

It can be observed that the volume of the ellipsoids for processing modes CPU and DirectML did not differ between the 100 body tracking runs and, therefore, had a standard deviation of 0.0 mm^3^ for all joints. The minimal ellipsoid volume for the processing mode CPU was 2.2 mm^3^ (SPINE_NAVEL), and the maximal ellipsoid volume was 1797.0 mm^3^ (FOOT_LEFT). The ellipsoid volume for the processing mode DirectML was between 1.4 (SPINE_NAVEL) and 392.6 mm^3^ (FOOT_LEFT).

For processing mode CUDA, the minimal ellipsoid volumes were between 1.3 (PELVIS) and 75.7 mm^3^ (FOOT_RIGHT) and the maximum volumes were between 9.2 (SPINE_NAVEL) and 2546.2 mm^3^ (FOOT_LEFT). The means of the volumes were between 3.4 (PELVIS) and 513.1 mm^3^ (FOOT_LEFT), the medians were between 1.8 (PELVIS) and 232.1 mm^3^ (FOOT_RIGHT), and the standard deviations between 1.5 (SPINE_NAVEL) and 777.9 mm^3^ (FOOT_LEFT).

For processing mode TensorRT, the minimal ellipsoid volumes were between 1.4 (PELVIS) and 118.0 mm^3^ (FOOT_RIGHT) and the maximum volumes between 9.8 (SPINE_ NAVEL) and 2325.7 mm^3^ (FOOT_LEFT). The means of the ellipsoid volumes were between 6.2 (SPINE_NAVEL) and 1117.0 mm^3^ (FOOT_LEFT), and the medians between 6.1 (SPINE_NAVEL) and 291.2 mm^3^ (FOOT_LEFT). The standard deviations of the different volumes are between 2.4 (SPINE_NAVEL) and 1028.5 mm^3^ (FOOT_LEFT).

In general, the ellipsoid volume, i.e., the variations over time and different runs, were the largest for the processing modes CUDA and TensorRT. The variations in the outer extremities (elbows, wrists, knees, ankles, feet) were significantly higher than the variations in the upper body (PELVIS, spines, hips, shoulders) (Figure 6; Table A2).

**Figure 7:**
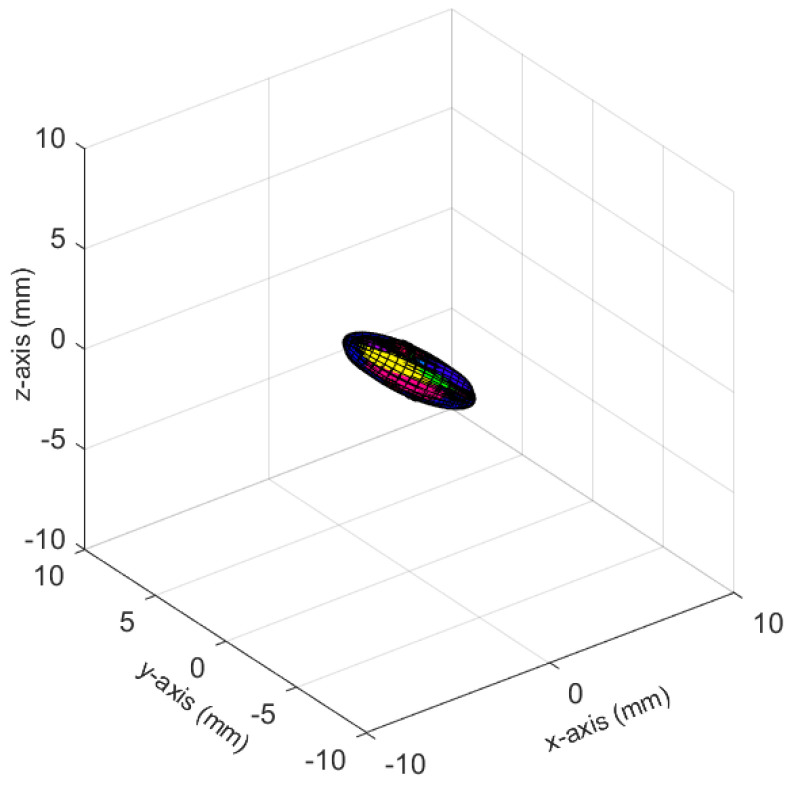
Ellipsoids of the joint position of the PELVIS for processing mode CUDA (Figures for all processing modes are shown in Figure A3). The different colors represent different body tracking runs.

**Figure 8:**
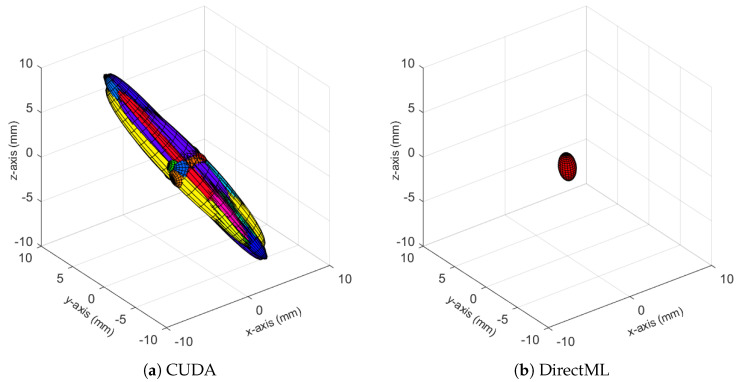
Ellipsoids of the joint position of KNEE_LEFT for relevant processing modes (Figures for all processing modes are shown in Figure A4). The different colors represent different body tracking runs.

**Figure 9:**
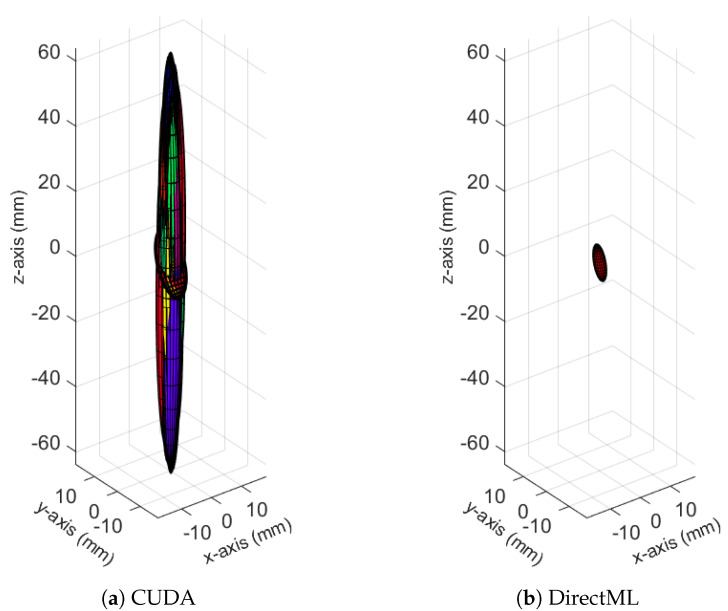
Ellipsoids for the joint position of FOOT_LEFT for relevant processing modes (Figures for all processing modes are shown in Figure A5). The different colors represent different body tracking runs.

The ellipsoids of the joints did not only differ in volume; their direction differed as well. The PELVIS showed very little variation over time, i.e., had a small ellipsoid volume. Its variations were mainly in the *x*-*y*-plane (see Figure 1a for reference), as shown for processing mode CUDA in Figure 7. The KNEE_LEFT mainly showed variations over time in the *y*-*z*-plane (Figure 8a, shown for processing mode CUDA), except for DirectML (Figure 8b), which showed little variation in all directions. FOOT_LEFT showed very large variations in the *z*-direction and much smaller variations in the *x*- and *y*-directions for all processing modes (Figure 9a, as shown for CUDA), except for DirectML (Figure 9b), which showed relatively little variation in the *z*-direction. We visually analyzed the directions of the ellipsoid’s semi-axes; however, from this analysis, no clear pattern emerged. The ellipsoids were neither clearly rotated in the direction of the camera’s laser rays nor distorted closer to the edge of the depth camera’s field of view.

**Figure 10:**
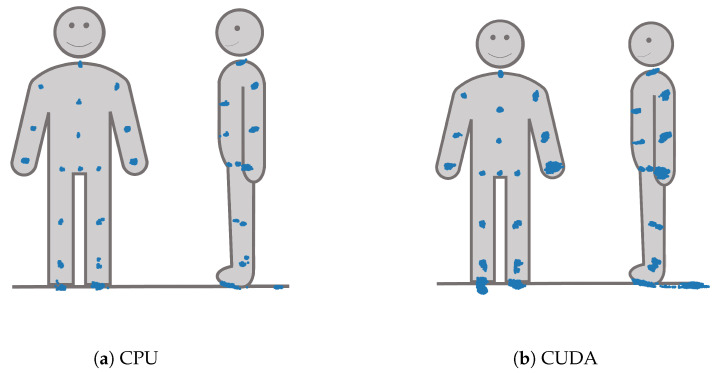

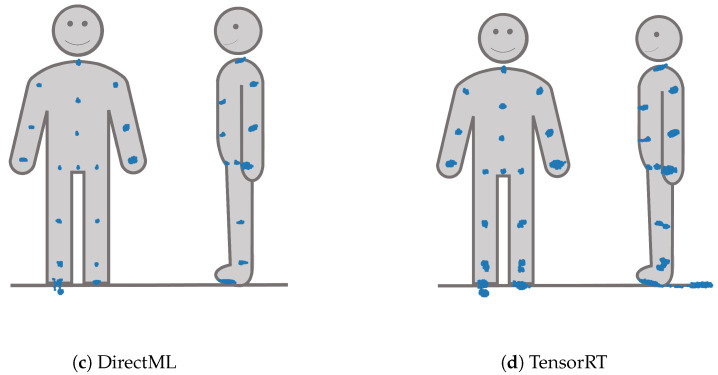
*X*-, *y*-, and *z*-*y*-plots of the joint positions seen from the frontal and left side perspective for all four processing modes.

The spatiotemporal distribution for each of the joints is visualized for each processing mode in Figure 10. It becomes clear that FOOT_LEFT had the biggest distribution in the *z*-direction. Furthermore, this visualization confirms that points of the outer extremities had a larger spatiotemporal distribution than the points of the upper body. Considering all joints, the processing mode DirectML overall had the smallest distributions, followed by CPU. CUDA had the biggest distributions of all processing modes, closely followed by TensorRT. Visual analysis of the closest point toward the camera for all joints showed that all points are behind the body surface. The only exception is WRIST_LEFT, where the foremost points lie slightly in front of or inside the body surface.

##### Euclidean Distances of Joint Positions between the Processing Modes

The Euclidean distance between frames in different body tracking runs within the same processing mode was calculated as a metric to quantify the differences in the joint position between each body tracking run. The processing modes CPU and DirectML produced the same result in each run, i.e., their Euclidean distance was 0.0 mm (Table A3).

**Figure 11:**
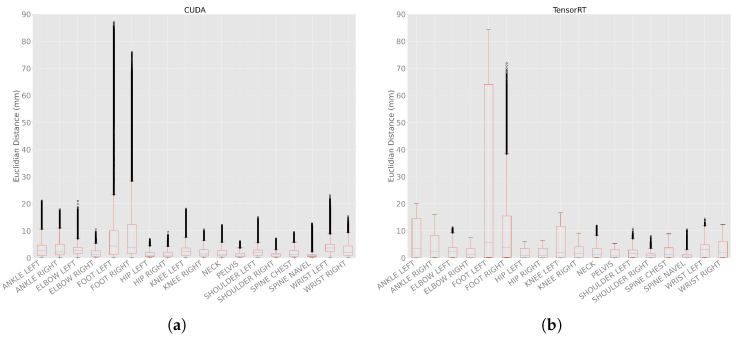
Euclidean distance with 100 body tracking runs using CUDA and TensorRT. (**a**) Euclidean distances with 100 body tracking runs using CUDA. (**b**) Euclidean distances with 100 body tracking runs using TensorRT.

For the processing mode CUDA (Figure 11a), the minimal Euclidean distance was 0.0 mm for all joints, and the maximum Euclidean distances were between 6.2 (PELVIS) and 87.2 mm (FOOT_LEFT). The means were between 0.9 (SPINE_NAVEL) and 17.9 mm (FOOT_LEFT), and the medians were between 0.7 (PELVIS) and 4.4 mm (FOOT_LEFT). The standard deviations were between 0.7 (SPINE_NAVEL) and 26.7 mm (FOOT_LEFT).

For processing mode TensorRT (Figure 11b), the minimal Euclidean distance was 0.0 mm for all joints as well. The maximum distances were between 5.3 (PELVIS) and 84.3 mm (FOOT_LEFT). The means were between 0.9 (SPINE_NAVEL) and 25.3 mm (FOOT_LEFT), and the medians between 0.7 (SPINE_NAVEL) and 5.7 mm (FOOT_LEFT). The standard deviations were between 0.9 (SHOULDER_RIGHT) and 30.7 mm (FOOT_LEFT).

Compared to CUDA (Figure 11a), TensorRT had wider confidence intervals (Figure 11b) with fewer outliers. Similar to the ellipsoid volume from Figure 6, it becomes clear that the Euclidean distance was higher for the outer extremities than for the upper body.

##### Variations in Bone Length between Body Tracking Runs

**Figure 12:**
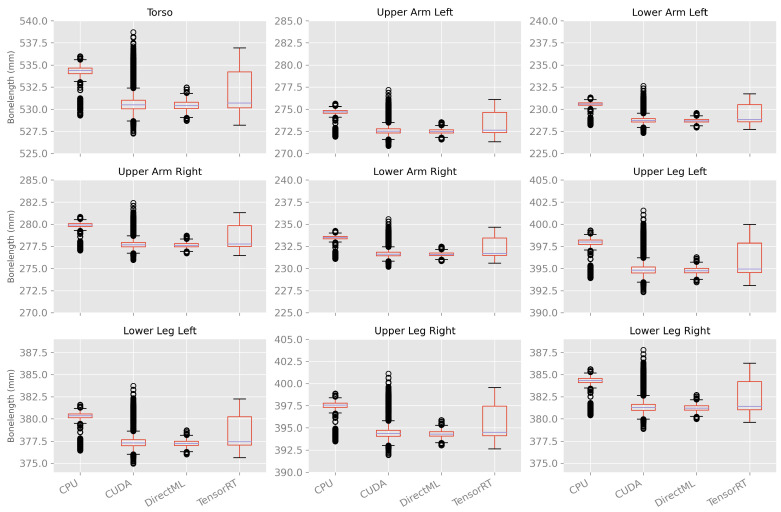
Box plots of bone length using 100 body tracking runs for all four processing modes.

The bone length was calculated as a metric for the differences in the size of the skeleton caused by the variations in body tracking between the various runs. As shown in Figure 12 and Table A4, the bone lengths varied much less over time and between the runs compared to the ellipsoid volume and Euclidean distance described above. In general, the mean bone lengths for processing modes CUDA, DirectML, and TensorRT were quite similar; the mean bone lengths for processing mode CPU were a few millimeters longer. DirectML showed the smallest standard deviation, followed by CPU and CUDA. TensorRT showed the largest standard deviations for the bone lengths but had no outlier.

### 3.3. Comparison of Different Computers

#### 3.3.1. Methods

Up to this point, we only considered the differences between body tracking runs on a single computer (computer A). Since we found substantial differences in the ellipsoid volumes, Euclidean distances, and bone lengths described above, we repeated the analyses on a second computer; computer B, described in Section 2.2.

To find similarities and differences between the results of computers A and B, the joint positions over time at the sensor’s *x*-, *y*-, and *z*-axes, as well as the calculated bone lengths from both computers, were compared.

#### 3.3.2. Results

**Figure 13:**
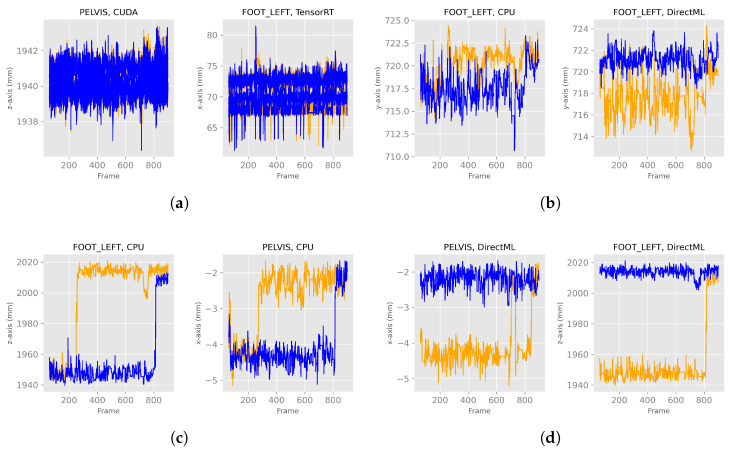
*X*-, *y*-, and *z*-axes of the joint positions of PELVIS and FOOT_LEFT for all four processing modes for two different computers—extract of relevant graphs (Figures for all processing modes and axis are shown in Figure A2). The blue lines represent computer A, and the orange ones computer B. (**a**) Examples of similar behavior between computers A and B. (**b**) Examples of very close steady value ranges for computers A and B. (**c**) Examples of a switch between two steady value ranges at different frames. (**d**) Examples of a switch between two steady value ranges just for one computer.

When looking at Figure 13a, one can see in these exemplary plots that for processing modes CUDA and TensorRT, the behaviors of computers A and B looked similar. Similar behavior was observed for both processing modes for the remaining axes and joints (Table 1). The behavior of the processing modes CPU and DirectML, on the other hand, were only partially similar between the two computers. For example, the *z*-axis of FOOT_LEFT in processing mode CPU showed a switch to another steady value range around frame 280 on computer B (Figure 13c). However, a similar switch on computer A was first observed around frame 800. DirectML switched between steady value ranges about 2 mm apart on the *x*-axis of the PELVIS and about 65 mm on the *z*-axis of the FOOT_LEFT on computer B, but both remained stable on computer A (Figure 13d). On the *y*-axis of FOOT_LEFT on processing mode CPU, the values on computer B were only slightly larger compared to computer A. Similar behavior was seen for DirectML (computer A slightly larger than computer B), although not entirely similar (Figure 13b). Here, the steady value ranges for both computers were not exactly the same but did not show clear, distinct differences compared to the steady value ranges in Figure 13c,d.

In general, the joint positions of the two computers differed for the processing modes DirectML and CPU, where the following distinct behaviors were observed: (1) one computer was continuously in the same steady value range, while the other computer switched to another steady value range (Figure 13d), (2) both computers switched, but at different points in time (Figure 13c), (3) the steady value ranges of both computers were very close to each other (Figure 13b).

**Figure 14:**
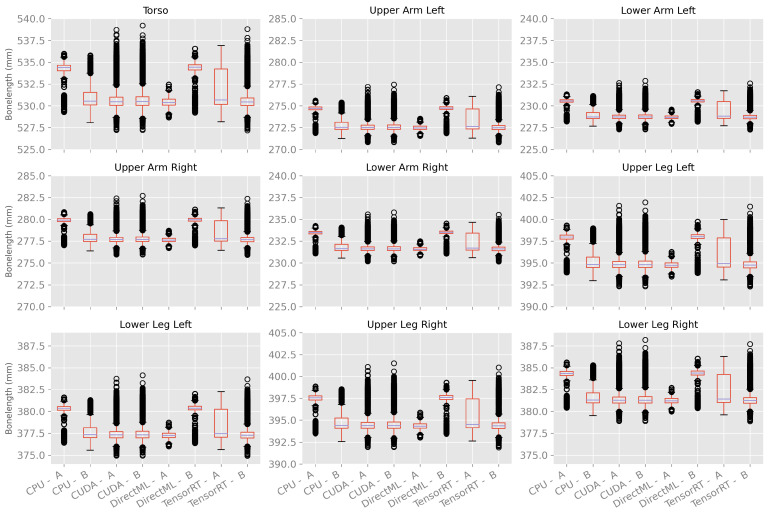
Box plots of bone length using 100 body tracking runs for all four processing modes on computers A and B.

The bone lengths showed similar properties for processing mode CUDA on both computers, as shown in Figure 14. For processing mode TensorRT, the bone lengths had a similar median. However, computer A had a bigger interquartile range as well as no outlier; computer B had a much smaller interquartile range and a lot of outliers. Thereby, TensorRT on computer B produced similar results compared to CUDA on computers A and B.

The processing modes CPU and DirectML, on the other hand, had a difference in the median. CPU on computer B and DirectML on computer A had a similar median to CUDA and TensorRT, and CPU on computer A and DirectML on computer B had an, on average, 2.7 mm higher median.

It is striking that the box plots of one computer and processing mode show the same pattern for all bone lengths (distance of the quartiles to the median, distance of the whiskers, and positioning of the outliers).

## 4. Discussion

The aim of this paper was to analyze and quantify the effects of the chosen processing mode on the results when using the Azure Kinect Body Tracking SDK. For this purpose, spatiotemporal changes in the joint positions, the differences within and among processing modes, as well as differences between two different computers were analyzed. We have shown that there were considerable differences between the processing modes, different runs of the body tracking and between different computers.

### 4.1. Consideration of Change over Time

In Section 3.1.2, we described a converging behavior of body tracking in the first seconds. To the best of our knowledge, this behavior of stabilization has not been described before. It seems to originate from some kind of initialization phase of body tracking. Similar behavior was observed when body tracking was started after the first 100 frames of the video instead of from the beginning, although the pattern seen was ambiguous. However, since the converging behavior seemed to be more pronounced when starting body tracking from the first frame, it could be that the depth sensor itself needs a few seconds to initialize or to focus. Furthermore, there might be some kind of pre- or post-processing in the body tracking that needs a few frames to stabilize. Unfortunately, the Azure Kinect Body Tracking SDK is closed-source and, therefore, a black box, so we can only speculate on the causes of the observed behavior. This phase of stabilization could be the subject of future work, whereby various possible camera settings, as well as external influences on the recording and body tracking, consequently make it difficult to analyze and isolate the reason for this stabilization phase.

Since the Azure Kinect Body Tracking SDK needs some time (around 60 frames @30 FPS) to stabilize in a position, we recommend starting body tracking from the first frame but waiting approximately two seconds (@30 FPS) before starting the subsequent analysis of the joint positions.

### 4.2. Comparison of Processing Modes

Our analyses in Section 3.2.2 showed that the spatiotemporal distribution of joint positions over all body tracking runs was the smallest using processing mode DirectML, followed closely by CPU, and larger for CUDA and TensorRT. One reason is that DirectML and CPU showed the same results for all 100 runs, while CUDA and TensorRT produced different joint positions in each run. Thus, DirectML and CPU, unlike CUDA and TensorRT, seem to yield reproducible data and thereby achieve results that are compatible with quality assurance and good scientific practice.

The Euclidean distances between the joint positions in all runs, as well as the volume of the ellipsoids, represent the spatiotemporal distribution of the joint positions over all body tracking runs and frames. We found that both were considerably higher for the outer extremities (especially feet, but also wrists) than for the upper body (PELVIS, spine, hips, shoulders). Our results are consistent with the results of Albert et al., who calculated the Euclidean distances between Azure Kinect DK body tracking and a Vicon system [10]. The increasing difference might be related to the fact that the skeleton of the Azure Kinect Body Tracking SDK is built up like a tree, with the PELVIS as its root and the feet, thumbs, hand tips, as well as the points in the face, as its leaves [18]. Consistently, the average errors of the outer extremities (feet, ankles, wrists, hands) were higher than those of the upper body (PELVIS, hips, spines, shoulders, clavicles). The speed of convergence in the first frames, on the other hand, seemed to be independent of the tree structure of the skeleton.

Furthermore, the body tracking seemed to stabilize in different steady value ranges; either it stayed in one steady range or it switched between different steady ranges. For CUDA and DirectML, less than half of the plots showed switches in the *x*-, *y*-, and *z*-axes. In contrast, these switches occurred in more than half of all plots for the processing modes CPU and TensorRT. Furthermore, the distribution of the switches over the frames varied for all processing modes. Therefore, it can be assumed that the switches were not caused by noise in the recorded video, but depended on the selected processing mode.

Moreover, it is noticeable that the distribution of the calculated bone lengths over the runs and frames showed a similar pattern for all bones. This suggests that the recognized skeleton probably has only minor changes in the size ratio of the individual bones and is merely scaled differently from frame to frame (and run to run). It is reasonable to assume that body tracking keeps some kind of (historical) skeletal model when detecting joint positions. Colombel et al. also suggest that the Azure Kinect Body Tracking SDK tracks individuals in an anatomically consistent manner as additional anthropomorphic constraints had only little effect on body tracking results [21]. It is also interesting that the maximum Euclidean distance between two runs was 87.2 mm (CUDA, FOOT_LEFT); the maximum difference in bone length, however, was only 11.5 mm (CUDA, Torso) and thus had significantly smaller variations.

All in all, when deciding which processing mode to use, one has to consider that CPU and DirectML had no differences in multiple body tracking runs on the same computer, whereas CUDA and TensorRT had differences of up to 87 mm. This means CPU and DirectML yielded seemingly consistent and repeatable results (important for quality assurance); in contrast, CUDA and TensorRT yielded inconsistent results. Besides that, one should be aware that the outer extremities have a higher spatiotemporal distribution than the upper body. For further analyses, such as body posture analyses or research involving humans, it must, therefore, be noted that the results become less accurate when focusing on the outer extremities compared to the upper body.

### 4.3. Comparison of Different Computers

Our results in Section 3.3.2 showed that CUDA and TensorRT produced similar results on both computers for both the spatiotemporal joint positions as well as the bone lengths. The processing modes CPU and DirectML, on the other hand, showed clear differences in the joint positions and bone lengths. We did not find any studies that compared body tracking between multiple computers.

One of the reasons that CUDA and TensorRT were similar on both computers could be based on the fact that they yielded different results between the runs, while DirectML and CPU yielded the same results in every run. As already described above, we observed different steady value ranges of joint positions. Multiple runs of CUDA and TensorRT covered several steady ranges. Conversely, CPU and DirectML covered only one of the steady ranges or switched from one steady range to another. As a result of this, the differences in CUDA and TensorRT between the two computers more or less averaged out, i.e., showed a regression to the mean. On the other hand, when the processing modes CPU or DirectML cover one steady range on one computer and another on the other computer (e.g., Figure 13c or Figure 13d), large differences in the joint position between the computers can occur. Consequently, this can result in larger differences in bone lengths. One should be aware of this phenomenon, therefore, we recommend performing all body tracking on a single computer.

### 4.4. Implications of the Results

Our results have shown that running body tracking repeatedly yielded clinically relevant differences in joint positions with Euclidean distances of up to 87 mm, depending on the processing mode used for the body tracking. Furthermore, we have shown that the computational hardware used can have an impact on the joint positions. Therefore, the results of previous studies using the Azure Kinect Body Tracking SDK might originate from differences in the body tracking algorithm instead of actual physiological effects being measured. Their results probably originate from a single body tracking run and might not be reproducible. It is difficult to assess the accuracy of their results since the processing mode used is usually not specified. As a consequence, the results from previous studies should be reevaluated, and until then, their findings should be interpreted with caution.

Not only the interpretation of previous studies is affected, also future studies need to take our results into account. The fact that body tracking might yield different results on multiple runs and with different processing modes implies that one should consider which data should be stored from a study. Several aspects should be taken into account: (i) data volume, (ii) privacy, and (iii) measurement error. Possible sets of data to store are: (a) raw data of the recorded video (RGB and depth); (b) raw data of the recorded video (depth only); (c) body tracking data of one run; or (d) body tracking data of multiple runs. Each of these sets has its specific (dis)advantages.

(a) The raw data requires a lot of storage space (i); the 30-s video used in this paper was 1.6 GB. At the same time, privacy (ii) is not assured since the subject can be identified from the video data. However, no erroneous data (iii) are stored, and body tracking can be executed again using future improved body tracking methods.
(b) When storing only the raw depth data, the data volume (i) is still high. However, the privacy (ii) of the subject is ensured a little better since it is more difficult to identify a person from depth data. Additionally, body tracking can be repeated at a later time (iii), as in (a).
(c) Storing only the body tracking data of one run requires the least amount of storage space (i) of all options. Privacy (ii) is ensured since no identifiable data is stored. However, differences between various body tracking runs, processing modes, and computers might strongly influence the result (iii).
(d) Body tracking data of multiple runs, on the other hand, require little storage space (i) and ensure privacy (ii). In addition, the measurement error could be reduced by aggregating or filtering the results from multiple body tracking runs (iii) to achieve reasonable accuracy. Although, it should be noted that aggregation and the filtering of body tracking runs are not trivial.

Saving a single body tracking run should normally suffice when the body tracking SDK yields reproducible results. While we have shown that this is not the case for the Azure Kinect Body Tracking SDK, we recommend saving the raw data or, when not possible, at least store multiple body tracking runs to ensure good scientific practice.

### 4.5. Limitations and Recommendations for Future Work

Our results showed that the processing modes of the Azure Kinect Body Tracking SDK introduce a number of anomalies. However, it should be noted that our study has several limitations. First, to exclude the influence of movements, we used a mannequin instead of a human. Although the mannequin was very well recognized by body tracking, it cannot be ruled out that body tracking of a real human differs. Second, for the same reason we analyzed a static scene; dynamic human movement might aggravate or reduce the differences found. Third, we analyzed a single pose; other poses (e.g., with self-occlusion) might produce different results. Fourth, we analyzed a single recording with a single frame rate and a single field of view setting to isolate the effects of the body tracking settings as much as possible. In future work, the findings presented in this paper should be confirmed for the other possible settings provided by the Azure Kinect (e.g., 15 FPS, 5 FPS, WFOV, binned versus unbinned). Fifth, we used the latest version (1.1.2) of the Azure Kinect Body Tracking SDK; other versions might exhibit different behavior. Sixth, we analyzed the differences in joint positions between the processing modes. However, it remains unknown which processing mode is the closest to the real joint positions. We recommend comparing the joint positions against the ground truth, e.g., obtained using the Vicon system similar to the experiments by Albert et al. [10] in future work. Although, one should be aware of the interference between the Azure Kinect and Vicon system when used simultaneously [14]. Seventh, we recorded the video used as an input for the body tracking in a windowless dark room to exclude influences of external light. That environment is not suitable for studies with human subjects. As already shown by Romeo et al. [13], ambient light can have an influence on body tracking. We recommend investigating the presented findings for different light conditions, preferably using illumination levels workable in studies with human subjects, and without infrared light.

## 5. Conclusions

This is, to the best of our knowledge, the first article that analyzed the differences in the Azure Kinect Body Tracking SDK between multiple runs, the four possible processing modes, and on different computers. We found substantial differences in body tracking results depending on the processing mode and computer used. The cause of these differences remains unclear because of the closed-source nature of the SDK. However, our results might have major consequences for all research performed using the Azure Kinect DK camera together with the Azure Kinect Body Tracking SDK since differences found in analyses of the body tracking might be caused by the processing mode instead of an actual physical effect on the measured subject.

To partially counteract these consequences or at least create awareness of the effects of the processing mode, we recommend the following for future studies that want to use the Azure Kinect DK (at least to evaluate static human poses):

- Be aware that running body tracking multiple times on the same recording might produce different results;
- Choose your processing mode wisely: CPU and DirectML seem to yield reproducible data (on the same computer), while CUDA and TensorRT do not;
- Report the processing mode in your publication;
- Do not start your analysis from the beginning of the body tracking, but skip a few frames (e.g., 60 frames) to let the joint positions converge to a steady state;
- Generate all body tracking results for your analyses on the same computer, since different computers result in different joint positions; and
- In case it is not possible to save the raw data of the recording (due to data volume constraints and/or privacy concerns), store multiple runs of body tracking data to reduce possible error effects.

## Figures and Tables

**Table 1:** Included and excluded joints from the Azure Kinect Body Tracking SDK joints in our analysis (for reference, see [18]).

Included Joints	Excluded Joints
PELVIS	CLAVICLE_LEFT
SPINE_NAVEL	HAND_LEFT
SPINE_CHEST	HANDTIP_LEFT
NECK	THUMB_LEFT
SHOULDER_LEFT	CLAVICLE_RIGHT
ELBOW_LEFT	HAND_RIGHT
WRIST_LEFT	HANDTIP_RIGHT
SHOULDER_RIGHT	THUMB_RIGHT
ELBOW_RIGHT	HEAD
WRIST_RIGHT	NOSE
HIP_LEFT	EYE_LEFT
KNEE_LEFT	EAR_LEFT
ANKLE_LEFT	EYE_RIGHT
FOOT_LEFT	EAR_RIGHT
HIP_RIGHT	
KNEE_RIGHT	
ANKLE_RIGHT	
FOOT_RIGHT	

**Figure A1:**
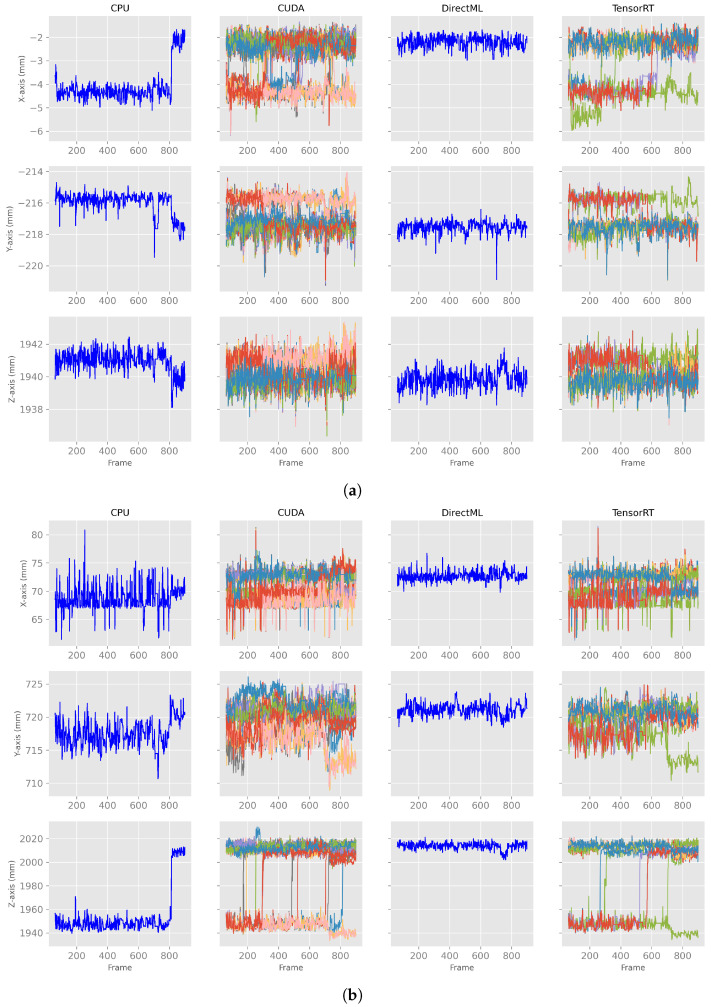
*X*-, *y*-, and *z*-axes of the joint positions of PELVIS and FOOT_LEFT for all four processing modes on computer A. (**a**) *X*-, *y*-, *z*-axes for the joint position of PELVIS. (**b**) *X*-, *y*-, *z*-axes for the joint position of FOOT_LEFT.

**Figure A2:**
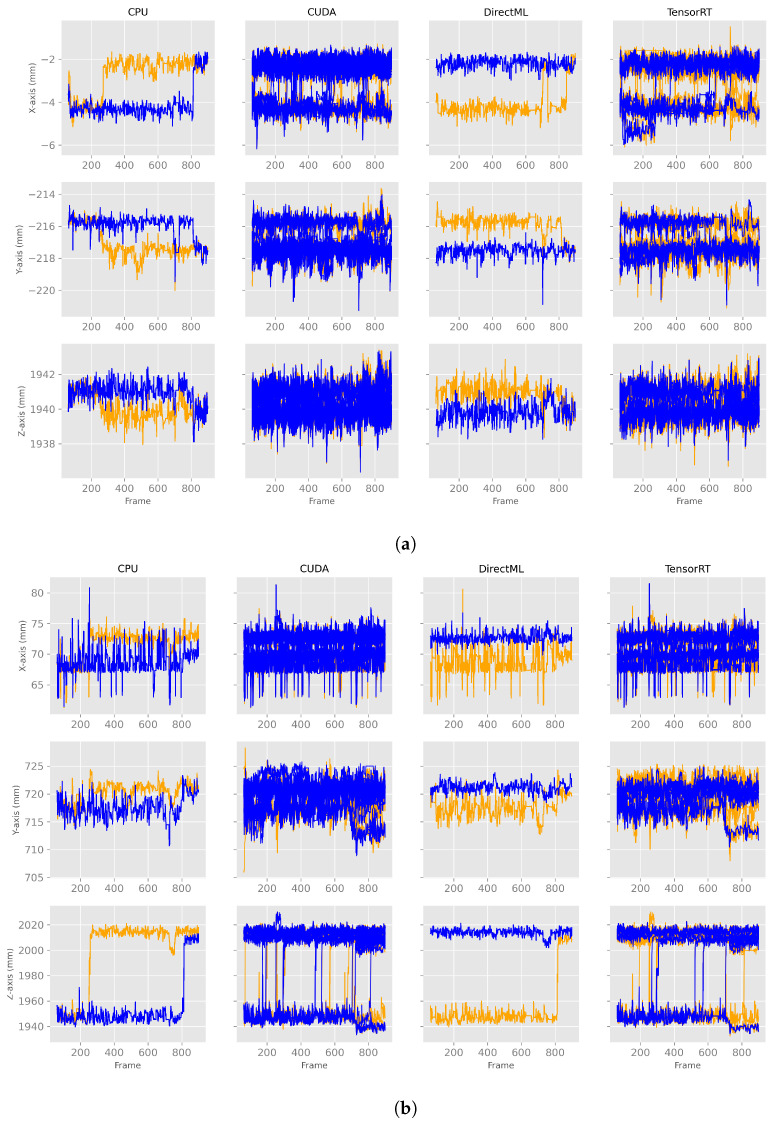
*X*-, *y*-, and *z*-axes of the joint positions of PELVIS and FOOT_LEFT for all four processing modes for two different computers. The blue lines are from computer A and the orange from computer B. (**a**) *X*-, *y*-, *z*-axes for the joint position of PELVIS. (**b**) *X*-, *y*-, *z*-axes for the joint position of FOOT_LEFT.

**Figure A3:**
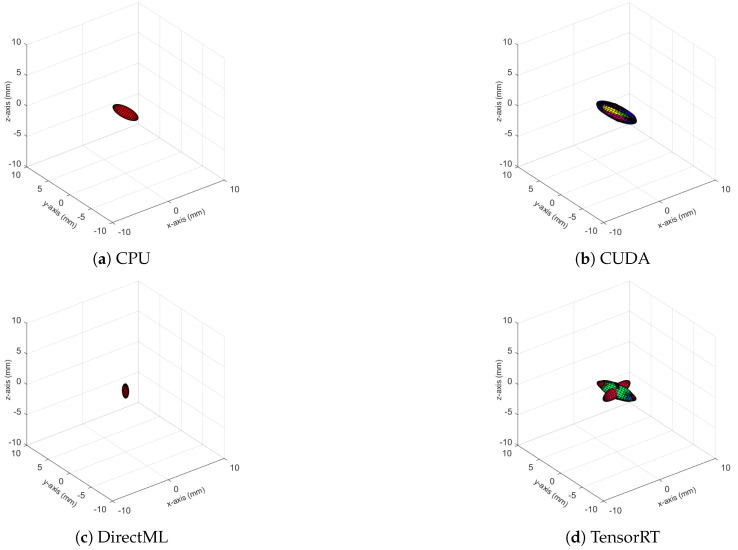
Ellipsoids of the joint position of PELVIS for all four processing modes. The different colors represent different body tracking runs.

**Figure A4:**
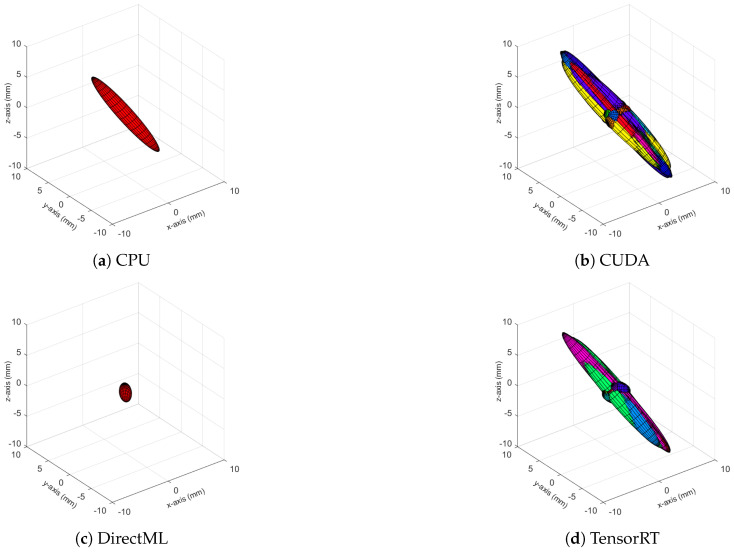
Ellipsoids of the joint position of KNEE_LEFT for all four processing modes. The different colors represent different body tracking runs.

**Figure A5:**
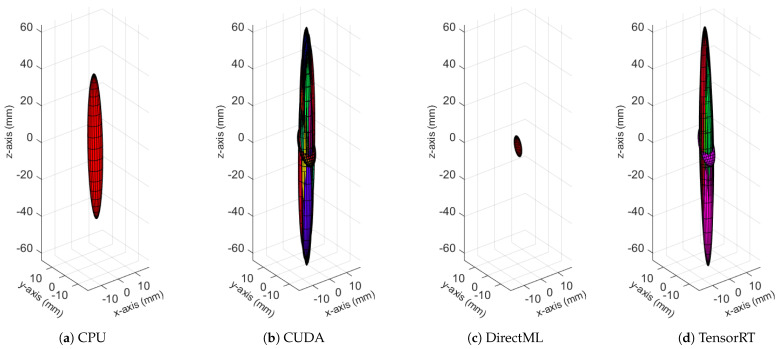
Ellipsoids for the joint position of FOOT_LEFT for all four processing modes. The different colors represent different body tracking runs.

## Data Availability

The data presented in this study are available on request from the corresponding author.

## Abbreviations

The following abbreviations are used in this manuscript:

CPU	Central Processing Unit
CUDA	Compute Unified Device Architecture
DirectML	Direct Machine Learning
DK	Developer Kit
FPS	Frames per Second
JSON	JavaScript Object Notation
MJPG	Motion JPEG
NFOV	Narrow Field of View
NPTS	Number of Faces of the Ellipsoid
RGB	Red Green Blue
RGB-D	Red Green Blue-Depth
RMSE	Root Mean Squared Error
SD	Standard Deviation
SDK	Software Development Kit
ToF	Time-of-Flight
WFOV	Wide Field of View

## Appendix A. Mapping between Bone Names and Joints

**Table A1:** Mapping between bone names and Azure Kinect joints.

Bone	Start Joint	End Joint
Torso	PELVIS	NECK
Upper Arm Left	SHOULDER_LEFT	ELBOW_LEFT
Lower Arm Left	ELBOW_LEFT	WRIST_LEFT
Upper Arm Right	SHOULDER_RIGHT	ELBOW_RIGHT
Lower Arm Right	ELBOW_RIGHT	WRIST_RIGHT
Upper Leg Left	HIP_LEFT	KNEE_LEFT
Lower Leg Left	KNEE_LEFT	ANKLE_LEFT
Upper Leg Right	HIP_RIGHT	KNEE_RIGHT
Lower Leg Right	KNEE_RIGHT	ANKLE_RIGHT

## Appendix C. Ellipsoid Volumes, Euclidean Distances and Bone Lengths

**Table A2:** Volume of the Ellipsoid containing 86.5% of all points for each combination of joint and processing mode. All values are in mm^3^.

Joint	Proc. Mode	Min	Max	Mean	Median	SD
ANKLE_LEFT	CPU	58.57	58.57	58.57	58.57	0.00
	CUDA	3.73	437.42	60.74	20.60	96.28
	DirectML	6.39	6.39	6.39	6.39	0.00
	TensorRT	5.04	208.39	50.11	46.42	41.67
ANKLE_RIGHT	CPU	40.06	40.06	40.06	40.06	0.00
	CUDA	22.26	248.33	63.50	45.65	41.66
	DirectML	36.97	36.97	36.97	36.97	0.00
	TensorRT	24.45	141.19	61.46	63.88	19.33
ELBOW_LEFT	CPU	61.79	61.79	61.79	61.79	0.00
	CUDA	36.92	233.58	79.59	74.01	27.61
	DirectML	60.07	60.07	60.07	60.07	0.00
	TensorRT	60.80	114.97	84.43	95.43	16.90
ELBOW_RIGHT	CPU	19.60	19.60	19.60	19.60	0.00
	CUDA	7.01	36.73	13.83	9.52	8.13
	DirectML	8.35	8.35	8.35	8.35	0.00
	TensorRT	8.35	31.60	20.46	24.31	10.77
FOOT_LEFT	CPU	1797.07	1797.07	1797.07	1797.07	0.00
	CUDA	39.75	2546.20	513.06	112.56	777.91
	DirectML	59.46	59.46	59.46	59.46	0.00
	TensorRT	53.61	2325.67	1116.96	291.18	1028.45
FOOT_RIGHT	CPU	203.74	203.74	203.74	203.74	0.00
	CUDA	75.69	1438.20	418.62	232.07	346.39
	DirectML	392.61	392.61	392.61	392.61	0.00
	TensorRT	117.98	1162.23	319.07	276.23	201.39
HIP_LEFT	CPU	6.69	6.69	6.69	6.69	0.00
	CUDA	1.77	20.44	5.25	2.61	5.04
	DirectML	2.12	2.12	2.12	2.12	0.00
	TensorRT	2.28	21.82	10.39	11.34	5.18
HIP_RIGHT	CPU	9.06	9.06	9.06	9.06	0.00
	CUDA	1.98	20.93	5.51	2.89	5.10
	DirectML	2.11	2.11	2.11	2.11	0.00
	TensorRT	2.32	17.36	10.80	12.86	5.13
KNEE_LEFT	CPU	42.63	42.63	42.63	42.63	0.00
	CUDA	2.57	149.86	29.96	12.99	38.40
	DirectML	5.35	5.35	5.35	5.35	0.00
	TensorRT	5.43	112.08	35.89	16.86	26.06
KNEE_RIGHT	CPU	18.72	18.72	18.72	18.72	0.00
	CUDA	7.11	38.21	15.20	12.24	7.22
	DirectML	9.43	9.43	9.43	9.43	0.00
	TensorRT	7.89	31.13	19.97	19.78	5.79
NECK	CPU	14.12	14.12	14.12	14.12	0.00
	CUDA	7.54	36.96	17.66	15.89	5.79
	DirectML	13.43	13.43	13.43	13.43	0.00
	TensorRT	10.55	30.31	19.99	17.90	4.80
PELVIS	CPU	5.54	5.54	5.54	5.54	0.00
	CUDA	1.30	12.34	3.41	1.75	3.07
	DirectML	1.40	1.40	1.40	1.40	0.00
	TensorRT	1.42	11.20	6.74	8.16	3.22
SHOULDER_LEFT	CPU	27.21	27.21	27.21	27.21	0.00
	CUDA	9.14	84.51	29.56	25.61	12.37
	DirectML	22.59	22.59	22.59	22.59	0.00
	TensorRT	10.57	61.84	36.01	40.26	9.94
SHOULDER_RIGHT	CPU	5.39	5.39	5.39	5.39	0.00
	CUDA	3.34	22.68	9.39	8.61	3.20
	DirectML	7.80	7.80	7.80	7.80	0.00
	TensorRT	3.87	23.19	9.14	8.29	3.36
SPINE_CHEST	CPU	12.48	12.48	12.48	12.48	0.00
	CUDA	5.08	39.37	11.10	8.12	6.84
	DirectML	6.60	6.60	6.60	6.60	0.00
	TensorRT	6.97	29.36	15.26	12.49	6.35
SPINE_NAVEL	CPU	2.18	2.18	2.18	2.18	0.00
	CUDA	1.61	9.24	3.71	3.21	1.54
	DirectML	2.03	2.03	2.03	2.03	0.00
	TensorRT	2.45	9.83	6.20	6.06	2.35
WRIST_LEFT	CPU	111.31	111.31	111.31	111.31	0.00
	CUDA	70.04	991.23	194.15	170.33	125.52
	DirectML	186.09	186.09	186.09	186.09	0.00
	TensorRT	110.31	305.99	218.65	194.07	80.32
WRIST_RIGHT	CPU	101.37	101.37	101.37	101.37	0.00
	CUDA	28.93	166.39	61.85	41.62	43.38
	DirectML	35.26	35.26	35.26	35.26	0.00
	TensorRT	33.69	165.84	99.81	81.74	59.64

**Table A3:** Euclidean distances for each joint and processing mode. All values are in mm.

Joint	Proc. Mode	Min	Max	Mean	Median	SD
ANKLE_LEFT	CPU	0.0	0.00	0.00	0.00	0.00
	CUDA	0.0	21.19	4.81	2.71	5.54
	DirectML	0.0	0.00	0.00	0.00	0.00
	TensorRT	0.0	19.97	6.23	3.43	6.58
ANKLE_RIGHT	CPU	0.0	0.00	0.00	0.00	0.00
	CUDA	0.0	18.04	3.52	2.20	3.29
	DirectML	0.0	0.00	0.00	0.00	0.00
	TensorRT	0.0	16.00	4.07	2.36	4.10
ELBOW_LEFT	CPU	0.0	0.00	0.00	0.00	0.00
	CUDA	0.0	21.05	3.04	2.76	2.00
	DirectML	0.0	0.00	0.00	0.00	0.00
	TensorRT	0.0	11.33	2.45	2.45	2.02
ELBOW_RIGHT	CPU	0.0	0.00	0.00	0.00	0.00
	CUDA	0.0	10.58	1.61	1.17	1.28
	DirectML	0.0	0.00	0.00	0.00	0.00
	TensorRT	0.0	7.42	1.71	1.15	1.62
FOOT_LEFT	CPU	0.0	0.00	0.00	0.00	0.00
	CUDA	0.0	87.18	17.85	4.43	26.73
	DirectML	0.0	0.00	0.00	0.00	0.00
	TensorRT	0.0	84.33	25.28	5.69	30.66
FOOT_RIGHT	CPU	0.0	0.00	0.00	0.00	0.00
	CUDA	0.0	76.08	8.43	3.75	11.78
	DirectML	0.0	0.00	0.00	0.00	0.00
	TensorRT	0.0	71.89	8.79	3.97	11.34
HIP_LEFT	CPU	0.0	0.00	0.00	0.00	0.00
	CUDA	0.0	6.96	1.38	0.82	1.29
	DirectML	0.0	0.00	0.00	0.00	0.00
	TensorRT	0.0	6.11	1.65	0.89	1.62
HIP_RIGHT	CPU	0.0	0.00	0.00	0.00	0.00
	CUDA	0.0	9.64	1.48	0.85	1.43
	DirectML	0.0	0.00	0.00	0.00	0.00
	TensorRT	0.0	6.51	1.72	0.90	1.70
KNEE_LEFT	CPU	0.0	0.00	0.00	0.00	0.00
	CUDA	0.0	18.18	4.18	2.51	4.61
	DirectML	0.0	0.00	0.00	0.00	0.00
	TensorRT	0.0	16.67	5.00	1.98	5.57
KNEE_RIGHT	CPU	0.0	0.00	0.00	0.00	0.00
	CUDAB	0.0	10.59	1.95	1.45	1.60
	DirectMLB	0.0	0.00	0.00	0.00	0.00
	TensorRTB	0.0	9.16	2.21	1.55	2.11
NECK	CPU	0.0	0.00	0.00	0.00	0.00
	CUDA	0.0	12.18	1.83	1.33	1.47
	DirectML	0.0	0.00	0.00	0.00	0.00
	TensorRT	0.0	11.91	1.89	1.31	1.84
PELVIS	CPU	0.0	0.00	0.00	0.00	0.00
	CUDA	0.0	6.18	1.18	0.67	1.15
	DirectML	0.0	0.00	0.00	0.00	0.00
	TensorRT	0.0	5.34	1.42	0.71	1.42
SHOULDER_LEFT	CPU	0.0	0.00	0.00	0.00	0.00
	CUDA	0.0	15.07	2.19	1.96	1.59
	DirectML	0.0	0.00	0.00	0.00	0.00
	TensorRT	0.0	10.81	1.86	1.74	1.66
SHOULDER_RIGHT	CPU	0.0	0.00	0.00	0.00	0.00
	CUDA	0.0	7.20	1.16	1.06	0.79
	DirectML	0.0	0.00	0.00	0.00	0.00
	TensorRT	0.0	8.02	0.94	0.84	0.87
SPINE_CHEST	CPU	0.0	0.00	0.00	0.00	0.00
	CUDA	0.0	9.52	1.79	1.25	1.46
	DirectML	0.0	0.00	0.00	0.00	0.00
	TensorRT	0.0	8.87	1.95	1.32	1.85
SPINE_NAVEL	CPU	0.0	0.00	0.00	0.00	0.00
	CUDA	0.0	12.73	0.85	0.74	0.65
	DirectML	0.0	0.00	0.00	0.00	0.00
	TensorRT	0.0	10.54	0.88	0.68	0.92
WRIST_LEFT	CPU	0.0	0.00	0.00	0.00	0.00
	CUDA	0.0	23.08	3.96	3.63	2.45
	DirectML	0.0	0.00	0.00	0.00	0.00
	TensorRT	0.0	14.49	3.08	2.96	2.62
WRIST_RIGHT	CPU	0.0	0.00	0.00	0.00	0.00
	CUDA	0.0	15.47	2.85	2.02	2.33
	DirectML	0.0	0.00	0.00	0.00	0.00
	TensorRT	0.0	12.39	3.05	1.88	2.98

**Table A4:** Bone lengths for each joint and processing mode. All values are in mm.

Bone	Proc. Mode	Min	Max	Mean	Median	SD
Lower Arm Left	CPU	228.21	231.33	230.40	230.57	0.61
	CUDA	227.30	232.64	228.95	228.73	0.72
	DirectML	227.95	229.58	228.70	228.69	0.24
	TensorRT	227.72	231.73	229.33	228.81	0.97
Lower Arm Right	CPU	231.10	234.26	233.31	233.49	0.62
	CUDA	230.18	235.58	231.85	231.62	0.73
	DirectML	230.84	232.49	231.59	231.58	0.24
	TensorRT	230.60	234.66	232.23	231.71	0.98
Lower Leg Left	CPU	376.46	381.59	380.06	380.34	1.01
	CUDA	374.96	383.76	377.67	377.30	1.19
	DirectML	376.02	378.71	377.25	377.24	0.39
	TensorRT	375.64	382.25	378.29	377.45	1.60
Lower Leg Right	CPU	380.42	385.61	384.06	384.34	1.02
	CUDA	378.90	387.79	381.65	381.27	1.21
	DirectML	379.98	382.70	381.22	381.20	0.39
	TensorRT	379.59	386.27	382.27	381.42	1.62
Torso	CPU	529.29	535.96	534.00	534.39	1.29
	CUDA	527.25	538.71	530.95	530.49	1.54
	DirectML	528.68	532.45	530.41	530.38	0.55
	TensorRT	528.18	536.91	531.73	530.69	2.06
Upper Arm Left	CPU	271.91	275.62	274.51	274.71	0.73
	CUDA	270.82	277.18	272.79	272.52	0.86
	DirectML	271.59	273.54	272.48	272.47	0.28
	TensorRT	271.32	276.09	273.23	272.62	1.16
Upper Arm Right	CPU	277.05	280.83	279.70	279.91	0.74
	CUDA	275.95	282.42	277.95	277.67	0.88
	DirectML	276.73	278.71	277.64	277.62	0.29
	TensorRT	276.45	281.32	278.40	277.78	1.18
Upper Leg Left	CPU	393.91	399.28	397.68	397.98	1.05
	CUDA	392.34	401.55	395.18	394.79	1.25
	DirectML	393.45	396.27	394.75	394.72	0.41
	TensorRT	393.06	399.98	395.83	394.95	1.68
Upper Leg Right	CPU	393.49	398.85	397.25	397.55	1.05
	CUDA	391.92	401.12	394.76	394.37	1.25
	DirectML	393.03	395.85	394.32	394.30	0.41
	TensorRT	392.63	399.54	395.41	394.52	1.68
